# Condyloma acuminata management in female patients differs by specialty and sociodemographic factors

**DOI:** 10.1016/j.jdin.2026.06.017

**Published:** 2026-07-06

**Authors:** Lauren A. Ling, Grace Sheng, Ivy Lee, Loida Luna, Lobna Raya, Catherine Baker

**Affiliations:** a Tufts University School of Medicine, Boston, Massachusetts; b Department of Dermatology, Tufts Medical Center, Boston, Massachusetts

**Keywords:** anogenital warts, condyloma acuminata, dermatology, health care disparities, papillomaviridae infections, practice patterns

*To the Editor:* Condyloma acuminata (CA) is a manifestation of human papillomavirus that is commonly managed across specialties. Variations in care may influence disease outcomes and contribute to disparities.^1^ This study aimed to evaluate specialty-specific–management patterns and examine associations between sociodemographic factors and treatment strategies among female patients with CA.

A retrospective chart review was conducted for female patients aged ≥18 years who were diagnosed with CA at dermatology or gynecology clinics at a large academic center from 2020 to 2025. Data collected included demographics, insurance, treatments, biopsy rates, and follow-up. Descriptive and inferential statistics, including multinomial logistic regression, were performed using Stata (StataCorp), with significance defined as *P* < .05.

Among 148 patients, 67% were non-Hispanic White, 18% Black, 5% Asian, 5% Hispanic, and 4% non-Hispanic other. Gynecology managed a higher volume of CA cases overall. Dermatology significantly favored cryotherapy as a first-line treatment *P* = .000), while gynecology more often used trichloroacetic acid (*P* = .001). Patients seen by dermatology were more likely to receive procedural treatment (*P* = .037) and less likely to receive topical treatment (*P* = .039) compared with those seen by gynecology. Black patients were 78% less likely to receive procedural treatment (*P* = .013), 72% less likely to receive topical treatment (*P* = .023), and 70% less likely to undergo biopsy (*P* = .045) compared with White patients. Non-Hispanic other patients were 95% less likely to follow up compared with White patients (*P* = .010).

As reflected in Fig 1, dermatology and gynecology differed significantly in their first-line CA treatment, favoring cryotherapy and trichloroacetic acid respectively. These differences may reflect variations in specialty-specific training and clinical resources; however, dermatologists are trained to use chemical treatment agents, so lack of training does not explain the differences in treatment pattern.^2^^,^^3^ Moreover, the number of follow-up visits did not differ between gynecology and dermatology patients (2.69 and 2.89, respectively; *P* = .821).

**Fig 1:**
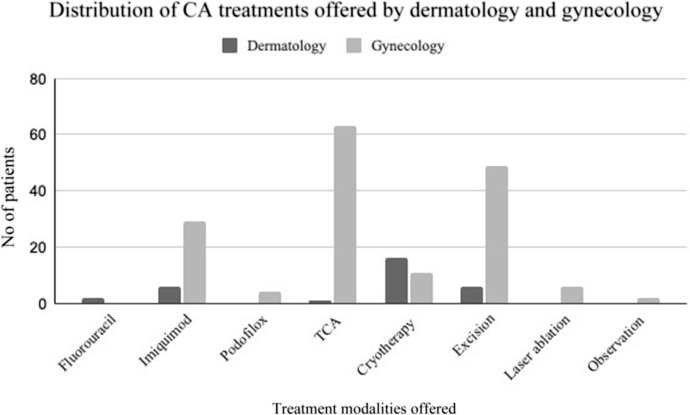
Distribution of condyloma acuminata (CA) treatments offered by dermatology versus gynecology. Dermatology favored cryotherapy as a first-line treatment for CA, while trichloroacetic acid (TCA) was the most used treatment in gynecology.

Racial and ethnic disparities in treatment and biopsy rates persisted independent of insurance status and specialty, suggesting that both provider practices and patient-level factors may contribute to inequities in care. Black patients were less likely to undergo procedural treatment, topical treatment, and skin biopsy compared with White patients. Furthermore, consistent with prior literature on general outpatient dermatology services, non-Hispanic other patients were found to have significantly fewer follow-up visits than White patients after adjusting for specialty and insurance status.^4^

Gynecology managed a higher volume of female patients with CA, as reflected in Table I. Comparable demographics across age, race, and insurance suggest that this distribution may be driven by factors such as provider availability and patient familiarity rather than underlying population differences. Most self-referrals were to gynecology, indicating that many patients view CA as a concern to discuss with their gynecologist.^5^ Ultimately, CA management is likely influenced by patient tolerance of procedures, thus making it difficult to assess specialty-based differences in treatment without taking patient preference into account.

**Table I:** Patient with condyloma acuminata demographics

Characteristic	Dermatology (*n* = 24)	Gynecology (*n* = 124)	Total (*n* = 148)	*P* value
Age (y), mean ± SD	44.71 ± 13.33	42.91 ± 13.39	43.20 ± 13.35	.55
Race/ethnicity				
White, non-Hispanic	18 (75.0%)	81 (65.3%)	99 (66.9%)	.75
Black, non-Hispanic	4 (16.7%)	23 (18.5%)	27 (18.2%)	
Asian, non-Hispanic	0 (0.0%)	8 (6.5%)	8 (5.4%)	
Other, non-Hispanic	1 (4.2%)	5 (4.0%)	6 (4.1%)	
Hispanic (any race)	1 (4.2%)	7 (5.6%)	8 (5.4%)	
Insurance status				
Private	16 (66.7%)	68 (54.8%)	84 (56.8%)	.44
Subsidized private	1 (4.2%)	14 (11.3%)	15 (10.1%)	
Public	7 (29.2%)	42 (33.9%)	49 (33.1%)	

While gynecology managed the majority of cases, patient demographics were similar across all specialties.

This study identified differences in CA management between dermatology and gynecology and across racial groups. Limitations include the small sample size and single-institution setting. Future multi-institutional studies should further evaluate structural and patient-focused determinants underlying these management differences, including cost, effectiveness, and patient satisfaction across treatment approaches.

## Conflicts of interest

None disclosed.
